# Transcriptomic Responses of *Mycoplasma bovis* Upon Treatments of *trans*-Cinnamaldehyde, Carvacrol, and Eugenol

**DOI:** 10.3389/fmicb.2022.888433

**Published:** 2022-06-06

**Authors:** Saurav Ranjitkar, Jingyue Ellie Duan, Kanokwan Srirattana, Fahad Alqahtani, Edan R. Tulman, Ion Mandoiu, Kumar Venkitanarayanan, Xiuchun Tian

**Affiliations:** ^1^Department of Animal Science, University of Connecticut, Storrs, CT, United States; ^2^National Center for Bioinformatics, King Abdulaziz City for Science and Technology, Riyadh, Saudi Arabia; ^3^Department of Pathobiology and Veterinary Science, University of Connecticut, Storrs, CT, United States; ^4^Department of Computer Science and Engineering, University of Connecticut, Storrs, CT, United States

**Keywords:** *trans*-cinnamaldehyde, carvacrol, eugenol, RNA, *Mycoplasma bovis*

## Abstract

*Mycoplasma bovis (M. bovis)* is an insidious, wall-less primary bacterial pathogen that causes bovine pneumonia, mid-ear infection, mastitis, and arthritis. The economic losses caused by *M. bovis* due to culling, diminished milk production, and feed conversion are underestimated because of poor diagnosis/recognition. Treatment with common antibiotics targeting the cell wall is ineffective. Plant-derived antimicrobials (PDAs) such as food-grade *trans*-cinnamaldehyde (TC), eugenol (EU), and carvacrol (CAR) are inexpensive and generally regarded as safe for humans and animals yet possess strong anti-bacterial properties. In preliminary studies, we found that all three PDAs inhibited the growth of *M. bovis in vitro*. Through RNA sequencing, we report here that CAR affected the expression of 153 genes which included the downregulation of energy generation-related proteins, pentose phosphate pathway, and upregulation of ribosomes and translation-related proteins. Few differentially expressed genes were found when *M. bovis* was treated with TC, EU, or when the three PDAs were double or triple combined. Our results suggest that, as opposed to the effect of CAR, the growth-inhibitory effects of TC and EU at levels tested may be exerted through mechanisms other than gene expression regulations.

## Introduction

Mycoplasmas are the smallest free-living bacteria and lack a cell wall (Razin, 1996). *Mycoplasma bovis* (*M. bovis*) is the most pathogenic mycoplasma in cattle (Caswell et al., 2010). It is well adapted to live on mucosal surfaces such as the respiratory tract and mammary glands (Peek et al., 2018). *M. bovis* can cause pneumonia, mastitis, mid-ear infection, arthritis, and other diseases. Cattle are most vulnerable during stressful conditions such as lactation and transport (Jasper et al., 1974; Arcangioli et al., 2008). The estimated losses caused by *M. bovis* from involuntary culling, reduced feed conversion and decreased production, medical treatments, extra labor, etc. are more than $2 billion/year worldwide (Maunsell et al., 2011). Outbreaks of *M. bovis* diseases have been reported in virtually every country, the most recent being New Zealand (July 21, 2017), a country with no prior records of *M. bovis* diseases according to their Ministry of Primary Industries (Boyce et al., 2021).

*M. bovis* is resistant to commonly used antibiotics because it lacks a cell wall, does not synthesize nucleotides or amino acids (Wormser and Hardy, 2005), and has an unusual form of RNA polymerase (Razin et al., 1998). Although *M. bovis* is susceptible to a group of uncommon and expensive antibiotics such as enrofloxacin (Rosenbusch et al., 2005), resistance is increasingly observed (Lysnyansky and Ayling, 2016). For example, Cai et al. (2019) found that the minimum inhibitory concentrations (MIC_50_) of chlortetracycline, oxytetracycline, tilmicosin, and tylosin were much higher in 2009 than the values found in 1990 demonstrating decreased susceptibility of *M. bovis* to antibiotics over time. Even resistance to enrofloxacin was also reported (Sulyok et al., 2017).

While several vaccines have been developed, most showed inconsistent effectiveness (Nicholas et al., 2002; Perez-Casal et al., 2017), which is attributed to the fast recombination of surface lipoproteins (Caswell and Archambault, 2007). The first commercial vaccines analyzed were Mycomune^®^ R (BIOMUNE Co., Lenexa, KS, United States) and Pulmo-Guard^™^ MpB (American Animal Health, Inc.). Both were shown to be ineffective against *M*. *bovis* colonization of the upper respiratory tracts or development of lesions caused by *M. bovis.* Their efficacies were shown to be 44% and less than 1%, respectively (Soehnlen et al., 2011). Furthermore, the two currently available vaccines, MpBGaurd and Myco-B One Dose (Dudek et al., 2021), contain two and three field isolates and their soluble antigens, respectively. Myco-B One Dose was claimed to prevent death and morbidity by only 10.4 and 15.2%, respectively.^1^ With the lack of effective vaccines (Gautier-Bouchardon et al., 2014; Yair et al., 2020), the use of alternative prevention and treatment regimens for *M. bovis* is therefore called for.

Plant-derived antimicrobials (PDAs) such as *trans*-cinnamaldehyde (TC), carvacrol (CAR), and eugenol (EU) are generally considered safe (GRAS) to humans/animals yet possess strong anti-bacterial and anti-fungal effects (Michiels et al., 2007). For instance, cinnamaldehyde and EU have antimicrobial effects in a wide range of bacteria that infect cattle such as *Mannheimia haemolytica, Escherichia coli, Staphylococcus aureus*, and *M. bovis* (Rajamanickam et al., 2019). All three PDAs have also been shown to be inhibitory on numerous species of fungi such as *Cryptococcus neoformans*, *Cryptococcus laurentii*, and *Microsporum gypseum* (Kumari et al., 2017; Michalczyk and Ostrowska, 2021).

Although not completely understood, the mechanisms of PDAs actions may include breaking down membrane integrity/increasing permeability, disrupting energy production, and other metabolic regulatory functions (Swamy et al., 2016). Whether these compounds modulate gene expressions of mycoplasma, however, has not been reported.

In preliminary studies, we found that, at low doses, TC (0.02%), EU (0.08%), and CAR (0.04%) caused growth inhibition of *M. bovis* in culture. This study was designed to delineate the molecular mechanisms for such inhibition by transcriptomic analysis. We found CAR exhibited ample effects on gene expression, while TC and EU might exert growth inhibition through other mechanisms than gene expression regulations.

## Materials and Methods

### Chemicals

All reagents were purchased from Sigma-Aldrich (Sigma-Aldrich, Inc., St. Louis, MO, United States) unless otherwise specified. Ten percent stock solutions of TC (C80687-25G, Lot # MKCD4749), CAR (W224502-100G-K, Lot # MKBW8250V), and EU (E51791-100G, Lot # STBG9481) were made using dimethyl sulfoxide as the vehicle and stored at 4°C until use.

### *Mycoplasma bovis* Culture, PDA Treatments, and RNA Extraction

*Mycoplasma bovis* strain *PG45* from ATCC (Manassas, VA) was grown for 24 h at 37°C to 1×10^8^ color changing units per milliliter in Fortified Commercial (FC) broth media supplemented with 20% horse serum. The *M. bovis* culture was aliquoted to 1 ml stock and frozen at −80°C until further use. On days of the treatments, one vial of the stock was thawed, added to 10 ml of culture medium, and cultured for 24 h at 37°C. This fresh culture was used directly in subsequent treatments.

To determine the effects of PDAs on *M. bovis* gene expression, CAR, EU, and TC were supplemented in culture media as shown in Table 1. Working concentration of PDAs was obtained by adding PDA stock solutions to *M. bovis* cultures. The treatment was conducted for 12 h at 37°C.

**Table 1:** PDA treatments of *Mycoplasma bovis* in culture.

	Treatment	Concentration (%, mM)
Negative control	No mycoplasma	–
Positive control	No PDA	–
Single PDA	TC	0.02% (1.6 mM)
	CAR	0.04% (2.6 mM)
	EU	0.08% (5.18 mM)
Double PDAs	TC + CAR	0.02% + 0.04%
	TC + EU	0.02% + 0.08%
	EU + CAR	0.08% + 0.04%
Triple PDAs	TC + CAR + EU	0.02% + 0.04% + 0.08%

The concentrations, treatment combinations, and durations (Table 1) were selected from preliminary experiments which all showed significant growth inhibition on *M. bovis in vitro*. Growth inhibition was determined by the color change of the media: growth of *M. bovis* was visually determined by phenol red in media changing from red to orange during pyruvate metabolism and media acidification, and growth inhibition was associated with lack of color change.

At the end of the treatment period, *M. bovis* pellets were collected by centrifugation at 10,000 g for 10 min at 4°C. Finally, 10 ml of TRIzol^™^ (Invitrogen, Carlsbad, CA, United States) was added to the pellet and stored at −80°C until RNA was extracted. The experiment was conducted three times.

RNA extraction from *M. bovis* samples was performed according to the standard TRIzol extraction protocol (Rio et al., 2010). To remove any potential DNA contamination, the extracted RNA was incubated with 7.5 U of DNase I (Qiagen, Hilden, Germany) for 15 min at room temperature. Then, RNA was precipitated again with 0.1 volume of 3M sodium acetate and 2.5 volumes of absolute ethanol, washed in 75% ethanol. The RNA was resuspended with 20 μl of RNase free-water and stored at −80°C until further analysis.

### RNA Sequencing

#### Total RNA Quality Control

Total RNA was quantified and purity ratios determined for each sample using the Nanodrop 2000 spectrophotometer (Thermo Fisher Scientific, Waltham, MA, United States). To assess RNA quality, total RNA was analyzed on the Agilent TapeStation 4,200 (Agilent Technologies, Santa Clara, CA, United States) using the RNA High Sensitivity assay and following the manufacturer’s protocol. RNA Integrity Numbers for all the samples were between 8.3 and 9.4.

#### Illumina-Compatible Transcriptome Library Preparation and Sequencing

Total RNA samples (250 ng of Qubit-quantified total RNA input) were prepared for prokaryotic transcriptome sequencing using the Zymo-Seq RiboFree Total RNA library preparation kit (Zymo Research, Irvine, CA, United States) following the manufacturer’s protocol. Libraries were quantified using the dsDNA High Sensitivity Assay for Qubit 3.0 (Life Technologies, Carlsbad, CA, United States); then, the library quality was validated for fragment length and adapter dimer removal using the Agilent TapeStation 4,200 D1000 High Sensitivity assay (Agilent Technologies, Santa Clara, CA, United States). Sample libraries were pooled and sequenced on illumina HiSeq 4,000 platform with 75 bp, pair-end read. On average, 7–10 M total reads were obtained per sample (Supplementary Table 1).

### Read Mapping, Annotation, and Enrichment

Raw reads from Illumina sequencing were processed through TrimGalore/0.6.5 (Krueger et al., 2021) to remove the adapter sequences and to improve the read quality. High-quality reads were aligned with the latest assembly for *M. bovis* PG45 (https://ftp.ncbi.nlm.nih.gov/genomes/all/GCF/000/183/385/GCF_000183385.1_ASM18338v1/), using bowtie2/2.3.5.1 (Langmead and Salzberg, 2012) with very sensitive-local flag (Supplementary Table 1). The average read counts and mapping rates were 4,547,069 and 99.17%, respectively. The resulting sequence alignment map files were converted to binary alignment map files using samtools. The read counts were generated using the htseq/0.11.0 package (Anders et al., 2015), normalized, and analyzed for differential expression using the DESeq2 (Love et al., 2014) package in R (R Core Team, 2021). With the normalized counts, principal component analysis (PCA) plots and Pearson correlation coefficient matrix were created using R software. Heat maps from the normalized counts were generated using Heatmapper (Babicki et al., 2016). Genes were considered to be differentially expressed (DEGs) if *P*-adjusted (*P*-adj) values were < 0.1 and Log_2_ Fold change (FC) was > 1 or below < −1, which means gene levels were either doubled or halved by the treatments. All other plots were generated using ggplot2 package in R. Gene Ontology (GO) enrichment and Kyoto Encyclopedia of Genes and Genomes (KEGG) were analyzed using the differentially expressed genes by the Gene Set Enrichment Analysis (GSEA, http://gseapro.molgenrug.nl/) with default settings.

## Results

### Transcriptomic Changes of *Mycoplasma bovis* by PDA Treatments

While all PDAs and combinations exhibited the ability to inhibit growth of *M. bovis* cultures, CAR induced significant and substantial changes in gene expression; TC, CAR + EU, and TC + EU induced few, while other treatments did not result in statistically significant changes in gene expression (Table 2; Supplementary Table 2). Out of a total of 875 annotated genes (including 179 hypothetical proteins) in *M. bovis*, CAR significantly upregulated 65 and downregulated 88 genes, accounting for approximately 17.48% of all annotated genes. Among these 153 genes, 117 were functionally annotated and 36 were hypothetical proteins. The treatments of EU, TC + CAR, and triple PDAs did not result in any detectable changes in gene expression. When treated with EU + CAR, 10 genes were upregulated out of which eight encoded annotated proteins. TC + EU modulated just two genes, of which one encoded an annotated protein. TC alone upregulated one gene which encoded a hypothetical protein (Table 2). These results suggest that, at the levels used, PDAs might have exerted growth-inhibitory effects through different mechanisms such as membrane function disruption. Antagonistic effects among PDAs might also have played a role on the regulation of gene expression even though all treatments inhibited growth (Michiels et al., 2007; Bassolé and Juliani, 2012). Interestingly, with the exception of CAR, when PDAs did elicit a gene expression change, it was always upregulation despite the overall growth inhibition. Of the relatively few (13) differentially expressed genes affected by PDA treatments other than CAR alone, four were hypothetical proteins possibly exerting functions yet to be identified.

**Table 2:** The numbers of up- and downregulated DEGs by each PDA or PDA combinations.

Treatment	Upregulated	Downregulated
CAR	65	88
EU	0	0
TC	1	0
TC + CAR	0	0
EU + CAR	10	0
TC + EU	2	0
TC + EU+ CAR	0	0

### Transcriptomic Changes of *Mycoplasma bovis* Upon CAR Treatment

**Figure 1:**
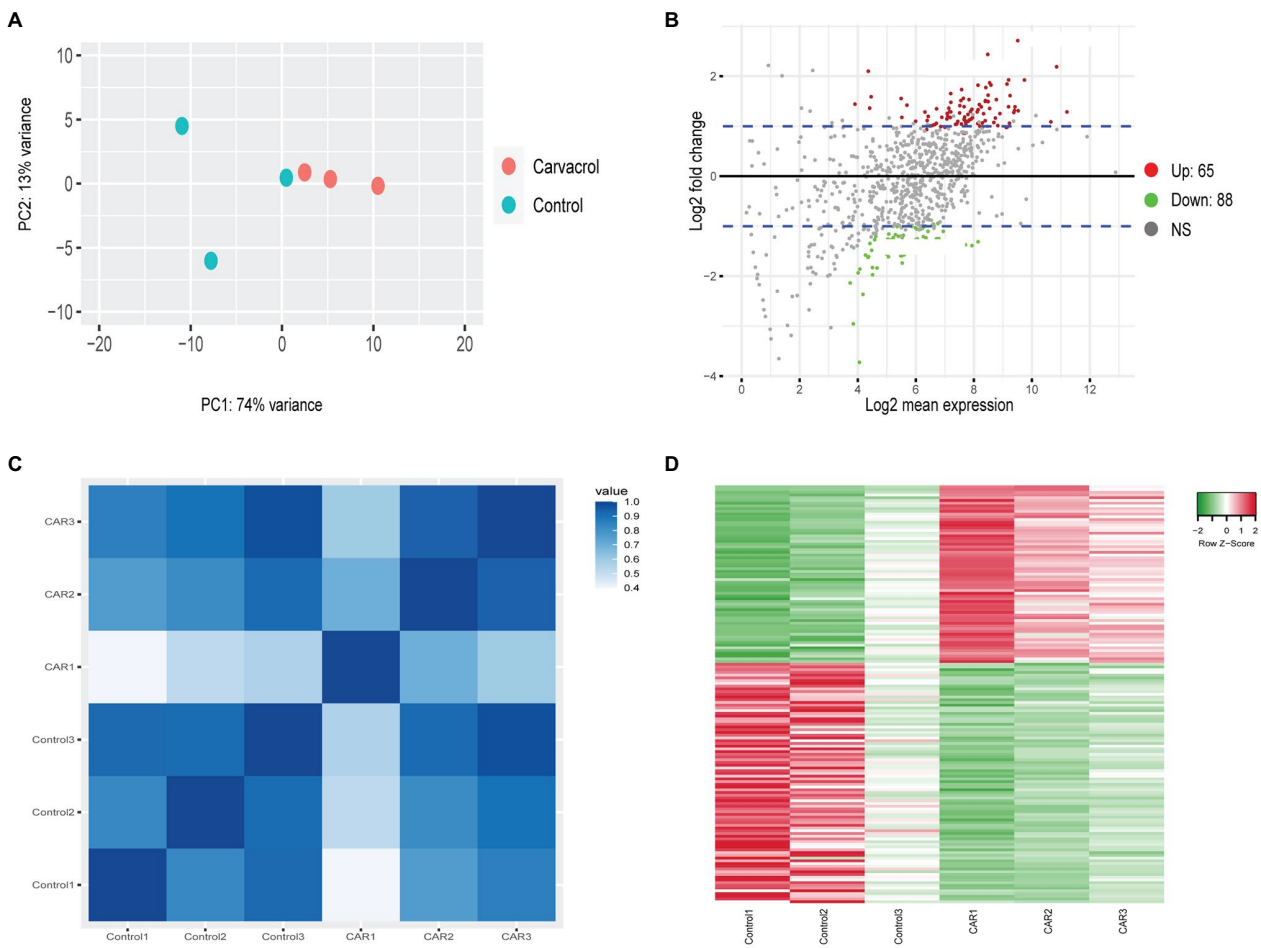
RNAseq analysis of Control vs. carvacrol (CAR) treated *Mycoplasma bovis.* **(A)** PCA of the transcriptomes for Control (green) and CAR (orange). Each dot represents a repeat of the experiments. **(B)** MA-plot (M = log ratio vs. A = mean average) showing upregulated (red), downregulated (green), and unchanged (gray) genes upon treatment of CAR. The plot was generated using an adjusted *p*-value of 0.1 and a Log_2_ fold change of 1. The blue lines denote Log_2_ fold change between 1 and −1. **(C)** Correlation plot between replicates of Control and CAR-treated samples. The color scale, ranging from white to blue, shows Pearson correlation coefficients ranging from 0.4 to 1, representing high to low correlation. **(D)** Heat map of Differentially expressed (DGE) gene between Control and CAR-treated samples. The color scale from green to red, corresponding with a z-score from −2 to 2, shows downregulated to upregulated genes. This clustering was acquired using average linkage and Pearson distance measurement method.

The PCA of Controls vs. PDA-treated samples did not generate any clustering except for those treated with CAR. This most likely resulted from the low number of DEGs in all treatments except for CAR. As shown in Figure 1, PC1 accounted for 74% of the variance between Control vs. CAR-treated samples, whereas PC2 explained 13% (Figure 1A). Two of the replicates for Control appeared to cluster together, whereas the third deviated from the Control group. On the other hand, replicates for CAR seemed to be grouped in the same area (Figure 1A). When *P*_adj_ and Log_2_FC were set to 0.1 and 1, respectively, genes whose levels were at least doubled or halved were regarded as up- and downregulated (blue lines in Figure 1B). Pearson correlation and heatmap analysis indicated that DEGs of the same treatment clustered together (Figures 1C,D).

Tables 3, 4 showed genes most down- and upregulated upon CAR treatment based on Log_2_FC. Interestingly, *M. bovis* growth inhibition by CAR was associated with upregulation of certain genes, suggesting that these genes might have been used to mitigate the adverse effects of CAR. Most genes upregulated in response to CAR were translation-related. Genes downregulated by CAR encoded variable surface lipoproteins, which may play role in pathogenicity, and metabolic genes. These data suggest that, in response to CAR, *M. bovis* increased growth-related functions like translation in attempt to counter growth inhibition. At the same time, the cells responded to CAR by reducing expression of genes associated with pathogenicity.

**Table 3:** The 12 most downregulated genes after CAR treatment based on Log_2_FC.

Genes	ID	*P*-adj	Log_2_FC
Hypothetical protein	MBOVPG45_RS04640	0.001	2.71
PTS transporter subunit EIIB	MBOVPG45_RS02840	0.001	2.44
Phosphoglycerate kinase	MBOVPG45_RS01105	0.006	2.19
Variable surface lipoprotein	MBOVPG45_RS04435	0.006	2.16
Hypothetical protein	MBOVPG45_RS01030	0.025	2.10
NADH-dependent flavin oxidoreductase	MBOVPG45_RS00040	0.001	1.93
Holliday junction resolvase *RecU*	MBOVPG45_RS01290	0.006	1.93
Serine--tRNA ligase	MBOVPG45_RS00305	0.003	1.87
rRNA pseudouridine synthase	MBOVPG45_RS01270	0.027	1.84
Lipoate--protein ligase A	MBOVPG45_RS01710	0.009	1.82
Nucleotidyltransferase	MBOVPG45_RS01710	0.001	1.82
BspA family leucine-rich repeat surface protein	MBOVPG45_RS02845	0.015	1.77

**Table 4:** The 12 most upregulated genes after CAR treatment based on Log_2_FC.

Genes	ID	*P*-adj	Log_2_FC
16S ribosomal RNA	MBOVPG45_RS01420	0.001	−3.72
16S ribosomal RNA	MBOVPG45_RS01410	0.006	−2.95
Hypothetical protein	MBOVPG45_RS04420	0.010	−2.36
Biotin/lipoyl-binding protein	MBOVPG45_RS01625	0.045	−2.14
50S ribosomal protein L6	MBOVPG45_RS01380	0.011	−1.97
30S ribosomal protein S18	MBOVPG45_RS02675	0.042	−1.93
F0F1 ATP synthase subunit B	MBOVPG45_RS02205	0.047	−1.86
F0F1 ATP synthase subunit A	MBOVPG45_RS02195	0.024	−1.84
Ribonuclease III	MBOVPG45_RS02585	0.020	−1.83
Hypothetical protein	MBOVPG45_RS00075	0.013	−1.74
Ribulose-phosphate 3-epimerase	MBOVPG45_RS03105	0.051	−1.62
50S ribosomal protein L3	MBOVPG45_RS01305	0.051	−1.61

**Figure 2:**
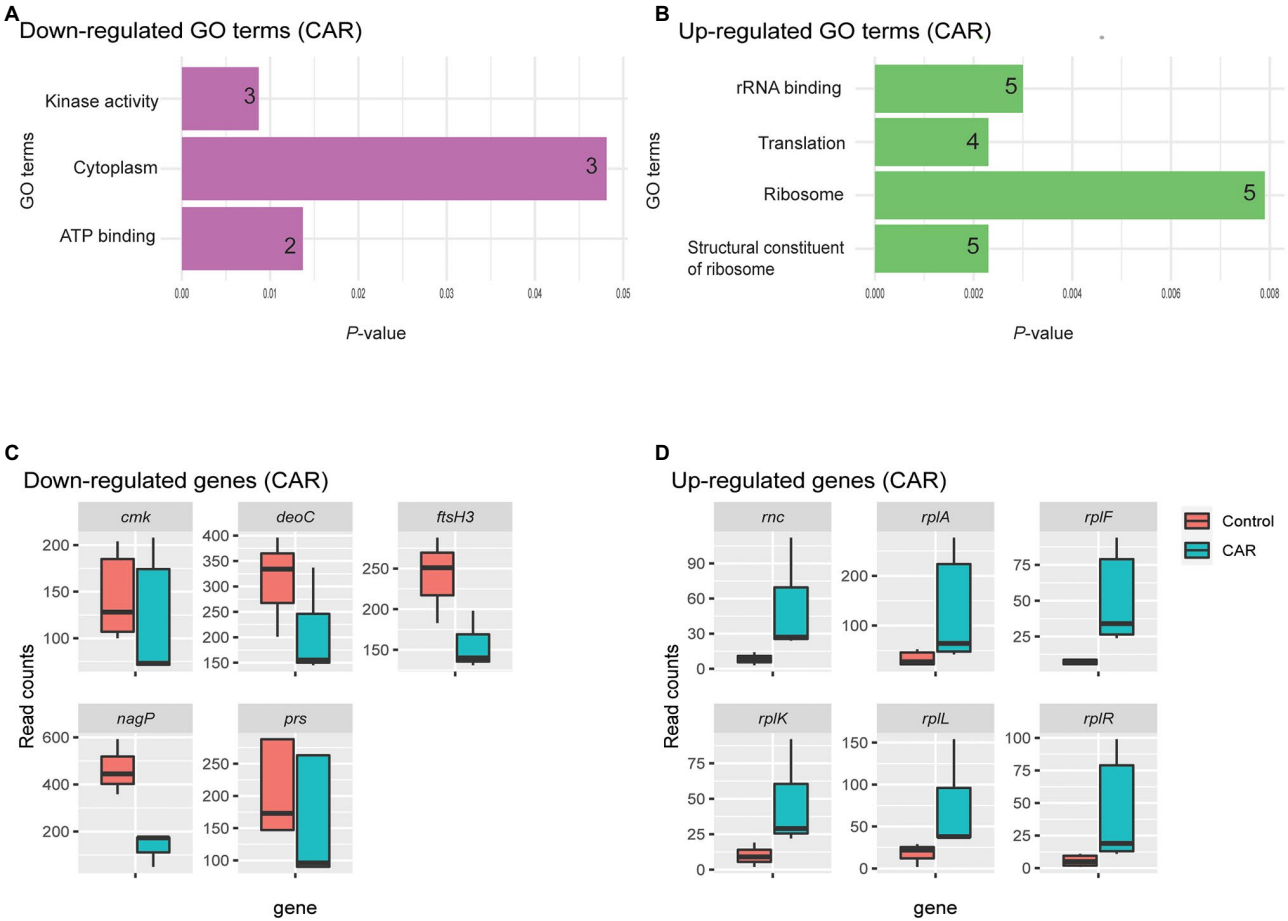
GO analysis of down- **(A)** and upregulated **(B)** genes by CAR treatment, plotted based on *p*-values. The number of genes within each term is noted in the bar. Upon CAR treatment, three and four significant GO terms were found among the down- and upregulated genes, respectively. Box plots of read counts for all genes down- **(C)** and upregulated **(D)** represented by the GO terms. While each GO term was represented by a number of genes, the total number of genes for the three downregulated GO terms was five and their read counts were plotted in C. The genes are as: Cytidylate kinase (*cmk*), deoxyribose-phosphate aldolase (*deoC*), ATP-dependent zinc metalloprotease (*ftsH3*), PTS system N-acetylglucosamine-specific EIICB (*nagP*), and ribonuclease 3 (*rnc*) and ribose-phosphate pyrophosphokinase (*prs*). Similar, six common genes represented the four upregulated GO terms plotted in D. They are ribonuclease 3 (*rnc*), 50S ribosomal protein L1(*rplA*), 50S ribosomal protein L6 (*rplF*), 50S ribosomal protein L11 (*rplK*), 50S ribosomal protein L7/L12 (*rplL*), and 30S ribosomal protein S18 (*rpsR*). Gene list with respect to their Go-Term terms is given in Supplementary Table 3.

Four GO terms were significantly associated with upregulated *M. bovis* genes upon CAR treatment: structural constituent of ribosome, ribosome, translation, and rRNA binding (Figure 2B). These four GO terms were represented by a total of six genes (Figure 2D). Structural constituent of ribosome, translation, and significant KEGG pathway ribosomal groups included genes like 50S ribosomal protein L11 (*rplK*), 50S ribosomal protein L1(*rplA*), 50S ribosomal protein L6 (*rplF*), 30S ribosomal protein S18 (*rpsR*), and 50S ribosomal protein L7/L12 (*rplL*). The ribosome category included *rplK*, *rplF*, *rpsR*, and *rplL*. The rRNA binding category included *rplA*, *rplF*, *rpsR*, and ribonuclease 3 (*rnc*) genes (Figure 2D).

### Transcriptomic Changes of *Mycoplasma bovis* Upon Other PDA Treatments

**Figure 3:**
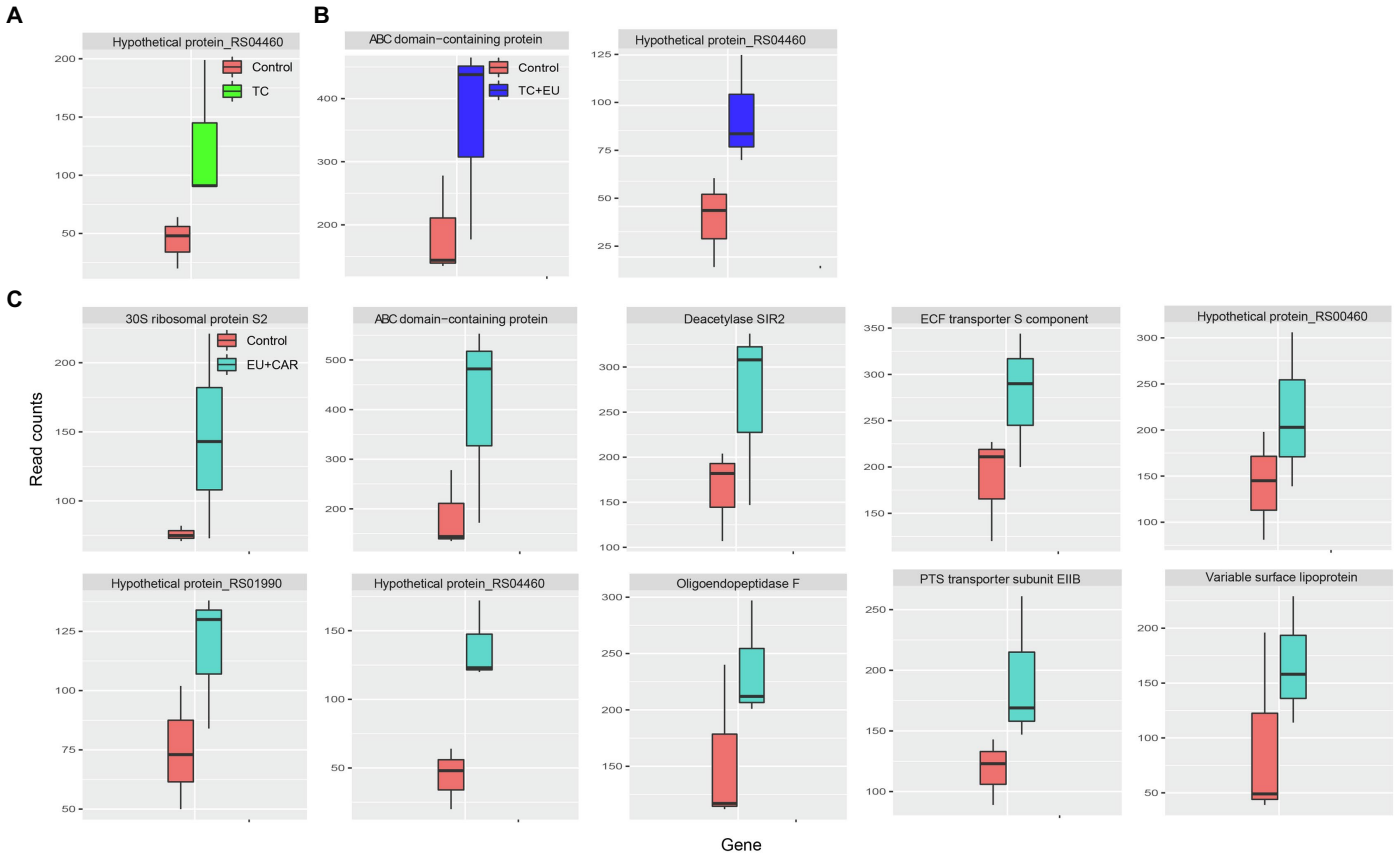
Box plots of read counts of upregulated genes after TC **(A)**, TC + EU **(B)**, and EU + CAR **(C)** treatments. The names of the genes are displayed on top of each panel (adjusted *p*-value <0.1).

Combined PDAs induced various responses of gene expression alternation. For example, when treated with EU + CAR, 10 genes were upregulated (Figure 3C), suggesting that EU might have reduced the effect of CAR since CAR treatment alone induced 153 DEGs. Alternatively, EU may have more rapidly exerted a growth-inhibitory effect than CAR, thus masking subsequent responses induced by CAR alone. Nonetheless, no significant GO term or KEGG pathway could be associated with the 10 genes differentially regulated after combined PDA treatments, most likely due to the low number of DEGs. TC + EU treatment induced two upregulated genes (Figure 3B), an ATP-binding cassette domain-containing protein and a hypothetical protein. TC-treated samples produced one upregulated gene (Figure 3A), encoding a hypothetical protein. Interestingly, we did not find any significantly downregulated genes when growth inhibition was induced with multiple-PDA treatments.

## Discussion

To our knowledge, this is the first transcriptomic study on *M. bovis*. While a previous study showed that EU and TC exhibited growth inhibition of *M. bovis in vitro* (Rajamanickam et al., 2019) which agreed with our preliminary growth inhibition results, the molecular mechanism of the PDA-mediated inhibition was not determined. Here, we showed that CAR’s inhibitory action on *M. bovis* involved differential expression of a large number of genes.

All PDAs used in our study have been linked with damage to cell membrane integrity, polarity, and free-radical generation in microbes (Devi et al., 2013; Shen et al., 2015; Al Wafai et al., 2017; Khan et al., 2017). It is therefore possible that membrane disruption may be the first action of PDAs at levels used in causing cell growth inhibition or cell death. Other actions such as altering gene expression regulation may be secondary. This could be the reason that we did not find large-scale gene expression changes for most PDA treatments (i.e., no DEGs from EU, TC + CAR, and Triple treatments, and very few from EU + CAR, TC + EU, and TC treatments).

A number of DEGs in CAR-treated samples are notable given their annotated function. A *BspA* family leucine-rich repeat surface protein is among the downregulated genes. *BspA* has been associated with *Tannerella forsythia* bacterial adhesion and invasion of epithelial cells *in vitro* (Inagaki et al., 2006). Downregulation of *BspA* upon CAR treatment weakens *M. bovis’* pathogenicity and may be a mechanism of CAR’s antimicrobial efficacy. Downregulation of *recU*, which plays an important role in chromosome segregation and DNA damage repair (Pereira et al., 2013), may be another potential mechanism for CAR’s antimicrobial action.

Nucleotidyltransferases are a family of enzymes that add nucleotides to substrates like nucleic acids and proteins (Neuenfeldt et al., 2008), and have involvement in DNA repairs (Liu et al., 2007). Upon CAR treatment nucleotidyltransferase is downregulated. This agrees with previous work which showed that the stress of 1,000 mg/l phenol decreased the expression of nucleotidyltransferase in *Acinetobacter strain Y* (Lin, 2017). Because CAR is a phenolic compound (Sharifi-Rad et al., 2018), this downregulation of nucleotidyltransferase is therefore a potentially logical mechanism for CAR’s antimicrobial effect by impairing DNA repair/synthesis. Another potential effect of CAR on the pathogenicity of *M. bovis* may be the downregulation of variable surface lipoproteins (Supplementary Table 2), which are assumed to be associated with the capacity of bacteria to adhere to epithelial cells and evade immune system (Christodoulides et al., 2018). This potential effect of CAR, although not related to growth inhibition, could significantly contribute to its overall antimicrobial effect (Michiels et al., 2007). Notably, combined EU + CAR treatment upregulated variable surface lipoprotein expression, but in genes paralogous to that affected by CAR treatment alone.

Phosphotransferase system (PTS) transporter subunit EIIB is part of the bacterial PTS system involved in uptake of sugar molecules like fructose, mannitol, and cellobiose (Deutscher et al., 2014). The downregulation of PTS by CAR treatment indicates disruption of *M. bovis* sugar uptake, which potentially reduces glycolysis and ATP generation. This is further exacerbated by the actual downregulation of ATP-binding proteins found in our GO analysis (Figures 2A,C). Gene *prs* is involved in synthesis of the intermediates for purine and pyrimidine, nicotinamide adenine dinucleotide synthesis (Hove-Jensen, 1988), whereas *ftsH3* involves in degradation of various transmembrane and cytoplasmic proteins (Srinivasan et al., 2008). Similarly, cytidylate kinase is one of the enzymes that are involved in nucleotide metabolism (An et al., 2014). This shows treatment with CAR results in disruption of cellular metabolism, including synthesis of DNA and RNA. Interestingly, when *M. bovis* was treated with EU + CAR, PTS transporter subunit EIIB expression was upregulated; this may be due to an antagonistic effect of CAR by EU (Bassolé and Juliani, 2012).

Moreover, we found CAR to affect expression of genes in the pentose phosphate pathway, including *deoC* and *prs* which were both downregulated by CAR. *deoC* is known to catalyze the reaction of 2-deoxy-D-ribose-5-phosphate into glyceraldehyde-3-phosphate and acetaldehyde (Haridas et al., 2018), while *prs* is the enzyme that generates phosphoribosyl diphosphate (Hove-Jensen et al., 2016). The inhibition of these enzymes conceivably reduces DNA replication and RNA synthesis, thus decreasing growth and replication of *M. bovis* in response to CAR.

Data here indicated that a major effect of CAR on *M. bovis* is to alter the translation process. However, CAR’s effect on translation-related genes was not uniform because different genes in the process could be either up- or downregulated. For example, serine- tRNA ligase and rRNA pseudouridine synthetase were downregulated by CAR, yet six ribosomal protein genes (*rplF, rpsR, rplK*, *rplA*, *rpsR*, and *rplL*; Kaczanowska and Rydén-Aulin, 2007) were upregulated, as was the pre-tRNA and rRNA processing enzyme *rnc* (Li, 2013). Similar conflicting effects of both up- and downregulation of different genes in the same pathways such as ATP generation (Guo et al., 2019; upregulation of ATP synthase subunits F0F1 ATP synthase subunit A and B) and pentose phosphate pathway (Lyngstadaas et al., 1998; upregulation of the ribulose-phosphate 3-epimerase) also occurred. These conflicting responses of *M. bovis* upon CAR treatment suggest that cells may have undergone chaotic changes of cellular processes and attempted to counter the reduction in growth by increasing protein translation and other inhibited processes.

Finally, we observed differential expression of many hypothetical proteins in response to CAR. Given the novelty of these proteins and their differential regulation in response to an inhibitory substance like CAR, they may be of great importance regarding survival or pathogenicity of *M. bovis* and represent important targets for direct experimentation in laboratory (Galperin, 2001). The identification of the functions of these hypothetical proteins will further our understanding of the effects of CAR.

In conclusion, at growth-inhibitory levels, TC, EU, and CAR exhibited different effects on gene expression by *M. bovis* with CAR inducing the most changes. It is likely that TC and EU at levels studied exerted their inhibitory effects through other mechanisms than that exerted by CAR. The antimicrobial effects of CAR involved the reduction of (1) certain translation-related enzymes, (2) DNA/RNA synthesis through pentose phosphorylation pathway, (3) surface proteins and lipoproteins associated with bacterial adhesion to and invasion of host tissues, and (4) ATP generation.

## Data Availability Statement

The datasets presented in this study can be found in online repositories. The names of the repository/repositories and accession number(s) can be found at: Gene Expression Omnibus (GEO)—GSE198086.

## Author Contributions

SR, JD, KS, IM, and FA conducted the experiments and/or data analysis. KV, ET, and XT designed the study. XT obtained research funding. All authors contributed to the drafting/editing of the manuscript. All authors contributed to the article and approved the submitted version.

## Funding

This study was supported by USDA grants to XT (58-8042-5-047 and W4171) and NSF grant to IM (1564936).

## Conflict of Interest

The authors declare that the research was conducted in the absence of any commercial or financial relationships that could be construed as a potential conflict of interest.

## Publisher’s Note

All claims expressed in this article are solely those of the authors and do not necessarily represent those of their affiliated organizations, or those of the publisher, the editors and the reviewers. Any product that may be evaluated in this article, or claim that may be made by its manufacturer, is not guaranteed or endorsed by the publisher.

## Footnotes

^1^ https://www.myco-b.com/new-2019-feedlot-study1.html

## References

[ref2] Al Wafai R. El-Rabih W. Katerji M. Safi R. El Sabban M. El-Rifai O.. (2017). Chemosensitivity of MCF-7 cells to eugenol: release of cytochrome-c and lactate dehydrogenase. Sci. Rep. 7:43730. doi: 10.1038/srep43730, PMID: 28272477 PMC5341120

[ref3] An J. H. Goo E. Kim H. Seo Y. S. Hwang I. (2014). Bacterial quorum sensing and metabolic slowing in a cooperative population. Proc. Natl. Acad. Sci. 111, 14912–14917. doi: 10.1073/pnas.1412431111, PMID: 25267613 PMC4205598

[ref4] Anders S. Pyl P. T. Huber W. (2015). HTSeq--a python framework to work with high-throughput sequencing data. Bioinformatics (Oxford, England) 31, 166–169. doi: 10.1093/bioinformatics/btu638, PMID: 25260700 PMC4287950

[ref5] Arcangioli M.-A. Duet A. Meyer G. Dernburg A. Bézille P. Poumarat F.. (2008). The role of Mycoplasma bovis in bovine respiratory disease outbreaks in veal calf feedlots. Vet. J. (London, England: 1997) 177, 89–93. doi: 10.1016/j.tvjl.2007.03.008 17493850

[ref6] Babicki S. Arndt D. Marcu A. Liang Y. Grant J. R. Maciejewski A.. (2016). Heatmapper: web-enabled heat mapping for all. Nucleic Acids Res. 44, W147–W153. doi: 10.1093/nar/gkw419, PMID: 27190236 PMC4987948

[ref7] Bassolé I. H. N. Juliani H. R. (2012). Essential oils in combination and their antimicrobial properties. Molecules 17, 3989–4006. doi: 10.3390/molecules17043989, PMID: 22469594 PMC6268925

[ref8] Boyce C. Jaye C. Noller G. Bryan M. Doolan-Noble F. (2021). Mycoplasma bovis in New Zealand: a content analysis of media reporting. Kōtuitui: New Zealand J. Social Sci. Online 16, 335–355. doi: 10.1080/1177083X.2021.1879180

[ref9] Cai H. Y. McDowall R. Parker L. Kaufman E. I. Caswell J. L. (2019). Changes in antimicrobial susceptibility profiles of mycoplasma bovis over time. Can. J. Vet. Res. 83, 34–41. 30670900 PMC6318825

[ref10] Caswell J. L. Archambault M. (2007). Mycoplasma bovis pneumonia in cattle. Anim. Health Res. Rev. 8, 161–186. doi: 10.1017/S1466252307001351 18218159

[ref11] Caswell J. L. Bateman K. G. Cai H. Y. Castillo-Alcala F. (2010). Mycoplasma bovis in respiratory disease of feedlot cattle. Vet. Clin. N. Am. Food Anim. Pract. 26, 365–379. doi: 10.1016/j.cvfa.2010.03.003 20619190

[ref12] Christodoulides A. Gupta N. Yacoubian V. Maithel N. Parker J. Kelesidis T. (2018). The role of lipoproteins in mycoplasma-mediated immunomodulation. Front. Microbiol. 9:1682. doi: 10.3389/fmicb.2018.01682, PMID: 30108558 PMC6080569

[ref13] Deutscher J. Aké F. M. D. Derkaoui M. Zébré A. C. Cao T. N. Bouraoui H.. (2014). The bacterial phosphoenolpyruvate: carbohydrate phosphotransferase system: regulation by protein phosphorylation and phosphorylation-dependent protein-protein interactions. Microbiol Mol Biol Rev 78, 231–256. doi: 10.1128/MMBR.00001-14, PMID: 24847021 PMC4054256

[ref14] Devi K. P. Sakthivel R. Nisha S. A. Suganthy N. Pandian S. K. (2013). Eugenol alters the integrity of cell membrane and acts against the nosocomial pathogen Proteus mirabilis. Arch. Pharm. Res. 36, 282–292. doi: 10.1007/s12272-013-0028-3, PMID: 23444040

[ref15] Dudek K. Szacawa E. Nicholas R. A. J. (2021). Recent developments in vaccines for bovine Mycoplasmoses caused by mycoplasma bovis and mycoplasma mycoides subsp. mycoides. Vaccine 9:549. doi: 10.3390/vaccines9060549, PMID: 34073966 PMC8225212

[ref16] Galperin M. Y. (2001). Conserved “hypothetical” proteins: new hints and new puzzles. Comp. Funct. Genomics 2, 14–18. doi: 10.1002/cfg.66, PMID: 18628897 PMC2447192

[ref17] Gautier-Bouchardon A. V. Ferré S. le Grand D. Paoli A. Gay E. Poumarat F. (2014). Overall decrease in the susceptibility of mycoplasma bovis to antimicrobials over the past 30 years in France. PLoS One 9:e87672. doi: 10.1371/journal.pone.0087672, PMID: 24503775 PMC3913625

[ref18] Guo H. Suzuki T. Rubinstein J. L. (2019). Structure of a bacterial ATP synthase. eLife 8:e43128. doi: 10.7554/eLife.43128, PMID: 30724163 PMC6377231

[ref19] Haridas M. Abdelraheem E. M. M. Hanefeld U. (2018). 2-Deoxy-d-ribose-5-phosphate aldolase (DERA): applications and modifications. Appl. Microbiol. Biotechnol. 102, 9959–9971. doi: 10.1007/s00253-018-9392-8, PMID: 30284013 PMC6244999

[ref20] Hove-Jensen B. (1988). Mutation in the phosphoribosylpyrophosphate synthetase gene (prs) that results in simultaneous requirements for purine and pyrimidine nucleosides, nicotinamide nucleotide, histidine, and tryptophan in *Escherichia coli*. J. Bacteriol. 170, 1148–1152. doi: 10.1128/jb.170.3.1148-1152.1988, PMID: 2449419 PMC210885

[ref21] Hove-Jensen B. Andersen K. R. Kilstrup M. Martinussen J. Switzer R. L. Willemoës M. (2016). Phosphoribosyl diphosphate (PRPP): biosynthesis, enzymology, utilization, and metabolic significance. Microbiol. Mol. Biol. Rev. (MMBR) 81, e00016–e00040. doi: 10.1128/MMBR.00040-16, PMID: 28031352 PMC5312242

[ref22] Inagaki S. Onishi S. Kuramitsu H. K. Sharma A. (2006). Porphyromonas gingivalis vesicles enhance attachment, and the Leucine-rich repeat BspA protein is required for invasion of epithelial cells by “Tannerella forsythia”. Infect. Immun. 74, 5023–5028. doi: 10.1128/IAI.00062-06, PMID: 16926393 PMC1594857

[ref23] Jasper D. E. Al-Aubaidi J. M. Fabricant J. (1974). Epidemiologic observations on mycoplasma mastitis. Cornell Vet. 64, 407–415. 4602678

[ref24] Kaczanowska M. Rydén-Aulin M. (2007). Ribosome biogenesis and the translation process in *Escherichia coli*. Microbiol Mol Biol Rev 71, 477–494. doi: 10.1128/MMBR.00013-07, PMID: 17804668 PMC2168646

[ref25] Khan I. Bahuguna A. Kumar P. Bajpai V. K. Kang S. C. (2017). Antimicrobial potential of Carvacrol against Uropathogenic *Escherichia coli* via membrane disruption, depolarization, and reactive oxygen species generation. Front. Microbiol. 8:2421. doi: 10.3389/fmicb.2017.02421, PMID: 29270161 PMC5724232

[ref27] Krueger F. James F. Ewels P. Afyounian E. Schuster-Boeckler B. (2021). FlixKrueger/TrimGalore: v0.6.7 - DOI via Zenodo (0.6.7). Zenodo doi: 10.5281/zenodo.5127899

[ref28] Kumari P. Mishra R. Arora N. Chatrath A. Gangwar R. Roy P.. (2017). Antifungal and anti-biofilm activity of essential oil active components against *Cryptococcus neoformans* and *Cryptococcus laurentii*. Front. Microbiol. 8:2161. doi: 10.3389/fmicb.2017.02161, PMID: 29163441 PMC5681911

[ref29] Langmead B. Salzberg S. L. (2012). Fast gapped-read alignment with bowtie 2. Nat. Methods 9, 357–359. doi: 10.1038/nmeth.1923, PMID: 22388286 PMC3322381

[ref30] Li Z. (2013). Pre-tRNA and pre-rRNA processing in bacteria. Encyclopedia Biol. Chem. 3, 554–560. doi: 10.1016/B978-0-12-378630-2.00277-2

[ref31] Lin J. (2017). Stress responses of Acinetobacter strain Y during phenol degradation. Arch. Microbiol. 199, 365–375. doi: 10.1007/s00203-016-1310-9, PMID: 27771745

[ref32] Liu Y. Prasad R. Beard W. A. Kedar P. S. Hou E. W. Shock D. D.. (2007). Coordination of steps in single-nucleotide base excision repair mediated by Apurinic/Apyrimidinic endonuclease 1 and DNA polymerase β *. J. Biol. Chem. 282, 13532–13541. doi: 10.1074/jbc.M611295200, PMID: 17355977 PMC2366199

[ref33] Love M. I. Huber W. Anders S. (2014). Moderated estimation of fold change and dispersion for RNA-seq data with DESeq2. Genome Biol. 15:550. doi: 10.1186/s13059-014-0550-8, PMID: 25516281 PMC4302049

[ref34] Lyngstadaas A. Sprenger G. A. Boye E. (1998). Impaired growth of an *Escherichia coli* rpe mutant lacking ribulose-5-phosphate epimerase activity. Biochim. Biophys. Acta 1381, 319–330. doi: 10.1016/s0304-4165(98)00046-4, PMID: 9729441

[ref35] Lysnyansky I. Ayling R. D. (2016). Mycoplasma bovis: mechanisms of resistance and trends in antimicrobial susceptibility. Front. Microbiol. 7:595. doi: 10.3389/fmicb.2016.00595, PMID: 27199926 PMC4846652

[ref36] Maunsell F. P. Woolums A. R. Francoz D. Rosenbusch R. F. Step D. L. Wilson D. J.. (2011). Mycoplasma bovis infections in cattle. J. Vet. Intern. Med. 25, 772–783. doi: 10.1111/j.1939-1676.2011.0750.x 21745245

[ref38] Michalczyk A. Ostrowska P. (2021). Essential oils and their components in combating fungal pathogens of animal and human skin. J. Med. Mycol. 31:101118. doi: 10.1016/j.mycmed.2021.101118, PMID: 33548912

[ref39] Michiels J. Missotten J. Fremaut D. de Smet S. Dierick N. (2007). In vitro dose–response of carvacrol, thymol, eugenol and trans-cinnamaldehyde and interaction of combinations for the antimicrobial activity against the pig gut flora. Livest. Sci. 109, 157–160. doi: 10.1016/j.livsci.2007.01.132

[ref40] Neuenfeldt A. Just A. Betat H. Mörl M. (2008). Evolution of tRNA nucleotidyltransferases: a small deletion generated CC-adding enzymes. Proc. Natl. Acad. Sci. 105, 7953–7958. doi: 10.1073/pnas.0801971105, PMID: 18523015 PMC2430343

[ref41] Nicholas R. A. J. Ayling R. D. Stipkovits L. P. (2002). An experimental vaccine for calf pneumonia caused by mycoplasma bovis: clinical, cultural, serological and pathological findings. Vaccine 20, 3569–3575. doi: 10.1016/s0264-410x(02)00340-7, PMID: 12297403 PMC7125750

[ref42] Peek S. F. Ollivett T. L. Divers T. J. (2018). “Respiratory diseases,” in Rebhun’s Diseases of Dairy Cattle (St. Louis: Elsevier), 94–167.

[ref43] Pereira A. R. Reed P. Veiga H. Pinho M. G. (2013). The Holliday junction resolvase RecU is required for chromosome segregation and DNA damage repair in *Staphylococcus aureus*. BMC Microbiol. 13:18. doi: 10.1186/1471-2180-13-18, PMID: 23356868 PMC3584850

[ref44] Perez-Casal J. Prysliak T. Maina T. Suleman M. Jimbo S. (2017). Status of the development of a vaccine against mycoplasma bovis. Vaccine 35, 2902–2907. doi: 10.1016/j.vaccine.2017.03.095, PMID: 28433326

[ref45] R Core Team (2021). R: A Language and Environment for Statistical Computing. Vienna, Austria: R Foundation for Statistical Computing.

[ref46] Rajamanickam K. Yang J. Sakharkar M. K. (2019). Phytochemicals as alternatives to antibiotics against major pathogens involved in bovine respiratory disease(BRD)and bovine mastitis(BM). Bioinformation 15, 32–35. doi: 10.6026/97320630015032, PMID: 31359996 PMC6651029

[ref47] Razin S. (1996). “Mycoplasmas,” in Medical Microbiology. 4th Edn. ed. Baron S.. (Galveston, TX: University of Texas Medical Branch at Galveston). 21413254

[ref48] Razin S. Yogev D. Naot Y. (1998). Molecular biology and pathogenicity of mycoplasmas. Microbiol. Mol. Biol. Rev. 62, 1094–1156. doi: 10.1128/MMBR.62.4.1094-1156.1998, PMID: 9841667 PMC98941

[ref49] Rio D. C. Ares M. Jr. Hannon G. J. Nilsen T. W. (2010). Purification of RNA using TRIzol (TRI reagent). Cold Spring Harb. Protoc. 2010:p.pdb.prot5439. doi: 10.1101/pdb.prot5439, PMID: 20516177

[ref50] Rosenbusch R. F. Kinyon J. M. Apley M. Funk N. D. Smith S. Hoffman L. J. (2005). In vitro antimicrobial inhibition profiles of mycoplasma bovis isolates recovered from various regions of the United States from 2002 to 2003. J. Vet. Diagn. Invest. 17, 436–441. doi: 10.1177/104063870501700505 16312234

[ref51] Sharifi-Rad M. Varoni E. M. Iriti M. Martorell M. Setzer W. N. del Mar Contreras M.. (2018). Carvacrol and human health: a comprehensive review. Phytother. Res.: PTR 32, 1675–1687. doi: 10.1002/ptr.6103, PMID: 29744941

[ref52] Shen S. Zhang T. Yuan Y. Lin S. Xu J. Ye H. (2015). Effects of cinnamaldehyde on *Escherichia coli* and *Staphylococcus aureus* membrane. Food Control 47, 196–202. doi: 10.1016/j.foodcont.2014.07.003

[ref53] Soehnlen M. K. Aydin A. Lengerich E. J. Houser B. A. Fenton G. D. Lysczek H. R.. (2011). Blinded, controlled field trial of two commercially available mycoplasma bovis bacterin vaccines in veal calves. Vaccine 29, 5347–5354. doi: 10.1016/j.vaccine.2011.05.092, PMID: 21664397

[ref54] Srinivasan R. Rajeswari H. Ajitkumar P. (2008). Analysis of degradation of bacterial cell division protein FtsZ by the ATP-dependent zinc-metalloprotease FtsH in vitro. Microbiol. Res. 163, 21–30. doi: 10.1016/j.micres.2006.03.001, PMID: 16638632

[ref55] Sulyok K. M. Kreizinger Z. Wehmann E. Lysnyansky I. Bányai K. Marton S.. (2017). Mutations associated with decreased susceptibility to seven antimicrobial families in field and laboratory-derived mycoplasma bovis strains. Antimicrob. Agents Chemother. 61, e01983–e01916. doi: 10.1128/AAC.01983-16, PMID: 27895010 PMC5278709

[ref56] Swamy M. K. Akhtar M. S. Sinniah U. R. (2016). Antimicrobial properties of plant essential oils against human pathogens and their mode of action: an updated review. Evid. Based Complement. Alternat. Med. 2016:e3012462, 1–21. doi: 10.1155/2016/3012462, PMID: 28090211 PMC5206475

[ref57] Wormser G. P. Hardy R. D. (2005). Mycoplasmas: molecular biology, pathogenicity, and strategies for controls edited by Alain Blanchard and Glenn Browning Norfolk, United Kingdom: horizon biosciences, 2005. 600 pp., illustrated. $179.95 (cloth). Clin. Infect. Dis. 41:1692. doi: 10.1086/497601

[ref58] Yair Y. Borovok I. Mikula I. Falk R. Fox L. K. Gophna U.. (2020). Genomics-based epidemiology of bovine mycoplasma bovis strains in Israel. BMC Genom. 21:70. doi: 10.1186/s12864-020-6460-0, PMID: 31969124 PMC6977290

