# Supplementary material for: Transcriptomic Responses of Mycoplasma bovis Upon Treatments of trans-Cinnamaldehyde, Carvacrol, and Eugenol
Source: Front Microbiol. 2022 Jun 6;13:888433. doi: 10.3389/fmicb.2022.888433 (PMC9207385; doi:10.3389/fmicb.2022.888433)
Supplement: Supplementary file 1 [file Table_1.docx]

**Supplementary Table 1. RNA-seq mapping results (against *M. bovis* PG45 reference genome)**

| **Samples** | **Number of reads** | **Number of uniquely mapped** | **Number of multiple mapped** | **Number of unmapped** | **Overall alignment (%)** |
| --- | --- | --- | --- | --- | --- |
| Control1 | 4,603,374 | 4,343,844 | 227,150 | 32,380 | 99.30 |
| Control2 | 5,082,262 | 4,745,370 | 306,435 | 30,458 | 99.40 |
| Control3 | 4,918,636 | 4,596,806 | 291,334 | 30,497 | 99.38 |
| TC1 | 3,120,084 | 2,440,553 | 596,949 | 82,582 | 97.35 |
| TC2 | 4,863,594 | 4,487,182 | 339,557 | 36,855 | 99.24 |
| TC3 | 4,496,607 | 4,126,377 | 331,104 | 39,126 | 99.13 |
| CAR1 | 4,641,334 | 4,342,501 | 263,703 | 35,130 | 99.24 |
| CAR2 | 4,289,930 | 3,938,804 | 320,265 | 30,862 | 99.28 |
| CAR3 | 4,182,130 | 3,850,509 | 301,382 | 30,240 | 99.28 |
| EU1 | 3,795,917 | 3,464,441 | 285,877 | 45,600 | 98.80 |
| EU2 | 5,187,812 | 4,887,469 | 261,606 | 38,738 | 99.25 |
| EU3 | 4,273,251 | 4,009,494 | 233,925 | 29,833 | 99.30 |
| EU+CAR1 | 4,733,964 | 4,265,595 | 422,343 | 46,027 | 99.03 |
| EU+CAR2 | 4,691,394 | 4,283,698 | 372,547 | 35,150 | 99.25 |
| EU+CAR3 | 4,909,653 | 4,448,396 | 426,312 | 34,946 | 99.29 |
| TC+CAR1 | 4,596,979 | 4,215,291 | 344,909 | 36,780 | 99.20 |
| TC+CAR2 | 5,060,841 | 4,659,745 | 365,466 | 35,631 | 99.30 |
| TC+CAR3 | 212,580 | 199,032 | 12,181 | 1,368 | 99.36 |
| TC+EU1 | 5,296,589 | 4,917,033 | 342,810 | 36,747 | 99.31 |
| TC+EU2 | 4,978,341 | 4,616,307 | 325,422 | 36,613 | 99.26 |
| TC+EU3 | 5,432,698 | 4,967,832 | 427,602 | 37,264 | 99.31 |
| Triple1 | 5,612,515 | 5,274,220 | 302,014 | 36,281 | 99.35 |
| Triple2 | 4,966,889 | 4,615,713 | 316,705 | 34,472 | 99.31 |
| Triple3 | 5,182,280 | 4,841,685 | 306,226 | 34,370 | 99.34 |
