# Supplementary material for: Transcriptomic Responses of Mycoplasma bovis Upon Treatments of trans-Cinnamaldehyde, Carvacrol, and Eugenol
Source: Front Microbiol. 2022 Jun 6;13:888433. doi: 10.3389/fmicb.2022.888433 (PMC9207385; doi:10.3389/fmicb.2022.888433)
Supplement: Supplementary file 3 [file Table_3.docx]

**Supplementary Table 3. Down- and up-regulated genes in GO terms of CAR treatment**

|  | **GO Term** | **Gene** | **Product** |
| --- | --- | --- | --- |
|  |  | *ftsH3* | ATP-dependent zinc metalloprotease |
|  | ATP binding | *prs* | Ribose-phosphate pyrophosphokinase |
|  |  | *cmk* | Cytidylate kinase |
|  |  |  |  |
|  |  | *deoC* | Deoxyribose-phosphate aldolase |
| Down-regulated | Cytoplasm | *prs* | Ribose-phosphate pyrophosphokinase |
|  |  | *cmk* | Cytidylate kinase |
|  |  |  |  |
|  | kinase activity | *nagP* | PTS system N-acetylglucosamine-specific EIICB component |
|  |  | *prs* | Ribose-phosphate pyrophosphokinase |
|  |  |  |  |
|  |  | *rplK* | 50S ribosomal protein L11 |
|  |  | *rplA* | 50S ribosomal protein L1 |
|  | Structural constituent of ribosome | *rplF* | 50S ribosomal protein L6 |
|  |  | *rpsR* | 30S ribosomal protein S18 |
|  |  | *rplL* | 50S ribosomal protein L7/L12 |
|  |  |  |  |
|  |  | *rplK* | 50S ribosomal protein L11 |
|  | Ribosome | *rplF* | 50S ribosomal protein L6 |
|  |  | *rpsR* | 30S ribosomal protein S18 |
|  |  | *rplL* | 50S ribosomal protein L7/L12 |
| Up-regulated |  |  |  |
|  |  | *rplK* | 50S ribosomal protein L11 |
|  |  | *rplA* | 50S ribosomal protein L1 |
|  | Translation | *rplF* | 50S ribosomal protein L6 |
|  |  | *rpsR* | 30S ribosomal protein S18 |
|  |  | *rplL* | 50S ribosomal protein L7/L12 |
|  |  |  |  |
|  |  | *rplA* | 50S ribosomal protein L1 |
|  | rRNA binding | *rplF* | 50S ribosomal protein L6 |
|  |  | *rnc* | Ribonuclease 3 |
|  |  | *rpsR* | 30S ribosomal protein S18 |
